# Supplementary material for: Engineering Peptide Inhibitors of the HFE–Transferrin Receptor 1 Complex
Source: Molecules. 2022 Oct 4;27(19):6581. doi: 10.3390/molecules27196581 (PMC9570809; doi:10.3390/molecules27196581)
Supplement: Supplementary file 1 [file molecules-27-06581-s001.zip › molecules-1853281-supplementary.pdf]

Supplementary Information for

Engineering peptide inhibitors of the HFE-Transferrin Receptor 1 Complex

Daniela Goncalves Monteiro[1], Gautam Rishi[2] Declan M. Gorman[1], Guillaume Burnet[1], Randy Aliyanto[1], K. Johan Rosengren[1], David M. Frazer[3], V. Nathan Subramaniam[2,*] and Richard J. Clark[1,*]

[1] The University of Queensland, School of Biomedical Sciences, Brisbane, QLD 4072, Australia
[2] School of Biomedical Sciences, Centre for Genomics and Personalised Health, Queensland University of Technology, Brisbane, QLD 4059, Australia
[3] QIMR Berghofer Medical Research Institute, 300 Herston Rd, Brisbane QLD 4006, Australia
*Correspondence: richard.clark@uq.edu.au (RJC); Nathan.subramaniam@qut.edu.au (VNS)

Contents:

- **Figure S1**. Analytical HPLC trace of TfR1 h1 with attached ESI-MS spectra. – page 2
- **Figure S2.** Analytical HPLC trace of TfR1 h3 with attached ESI-MS spectra. – page 3
- **Figure S3**. Analytical HPLC trace of TfR1 h1h3 with attached ESI-MS spectra. – page 4
- **Figure S4**. Analytical HPLC trace of TfR1 negative control with attached ESI-MS spectra. – page 5
- **Figure S5.** Analytical HPLC trace of HFEα1 with attached ESI-MS spectra. – page 6
- **Figure S6.** Analytical HPLC trace of HFEα2 with attached ESI-MS spectra. – page 7
- **Figure S7.** Analytical HPLC trace of HFEα1α2with attached ESI-MS spectra. – page 8
- **Figure S8.** Analytical HPLC trace of HFEα1α2 staple 1 with attached ESI-MS spectra. – page 9
- **Figure S9.** Analytical HPLC trace of HFEα1α2 staple 2 with attached ESI-MS spectra. – page 10
- **Figure S10**. Analytical HPLC trace of HFEα1α2 double staple with attached ESI-MS spectra. – page 11
- **Figure S11.** Analytical HPLC trace of HFE negative control with attached ESI-MS spectra. – page 12
- **Figure S12.** Analytical HPLC trace of HFEα1α2 scrambled sequence with attached ESI-MS spectra. – page 13

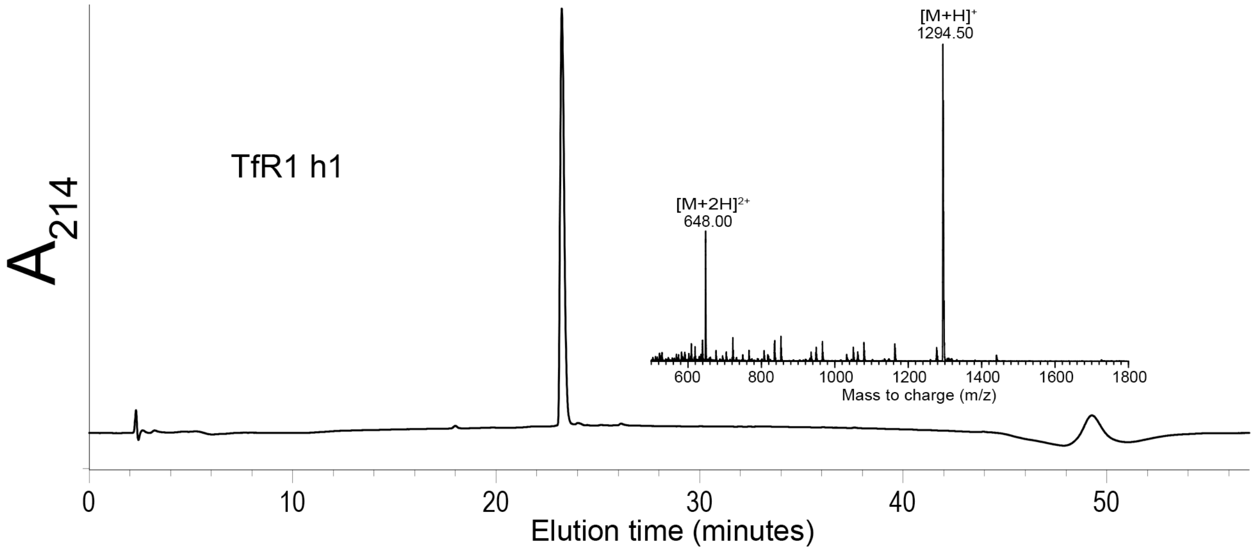

Figure S1. Analytical HPLC trace of TfR1 h1 with attached ESI-MS spectra. Analytical HPLC was run at an increasing gradient of 2% buffer B (90% ACN, 0.05% TFA) in buffer A (0.05% TFA) per minute using an Agilent, 300Å, 5 µm, 150 x 2.1 mm C18 column.

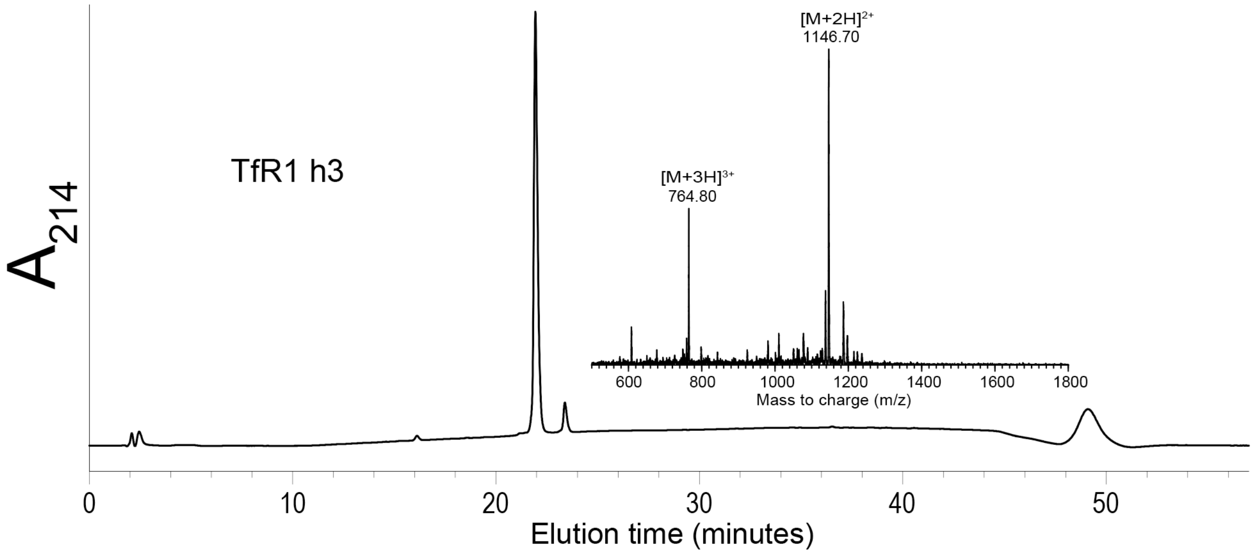

Figure S2. Analytical HPLC trace of TfR1 h3 with attached ESI-MS spectra. Analytical HPLC was run at an increasing gradient of 2% buffer B (90% ACN, 0.05% TFA) in buffer A (0.05% TFA) per minute using an Agilent, 300Å, 5 µm, 150 x 2.1 mm C18 column.

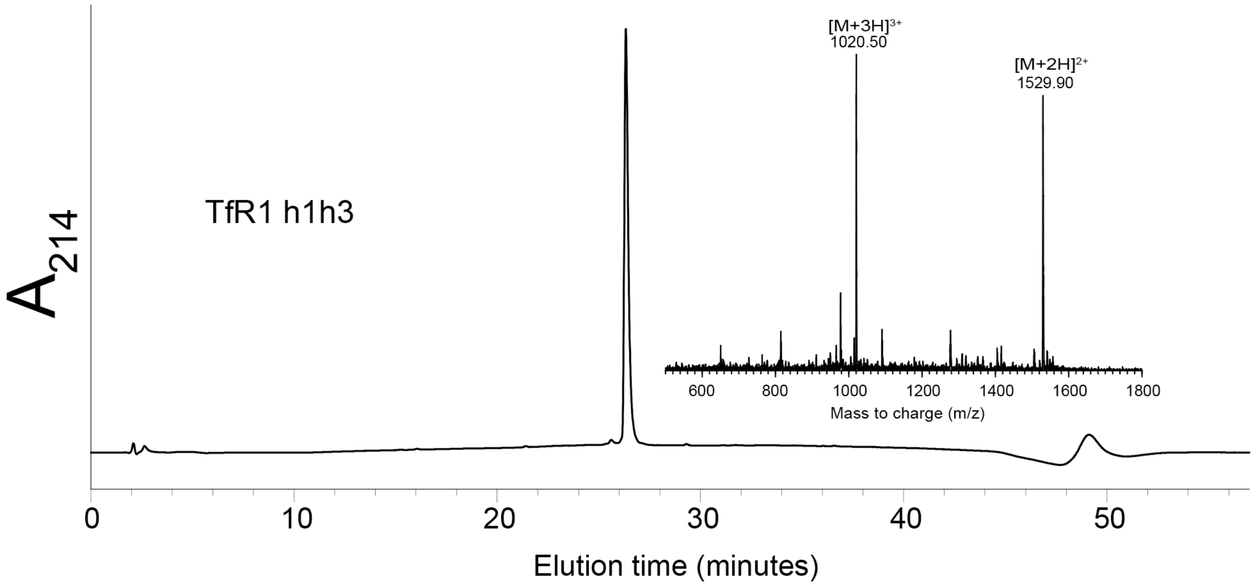

Figure S3. Analytical HPLC trace of TfR1 h1h3 with attached ESI-MS spectra. Analytical HPLC was run at an increasing gradient of 2% buffer B (90% ACN, 0.05% TFA) in buffer A (0.05% TFA) per minute using an Agilent, 300Å, 5 µm, 150 x 2.1 mm C18 column.

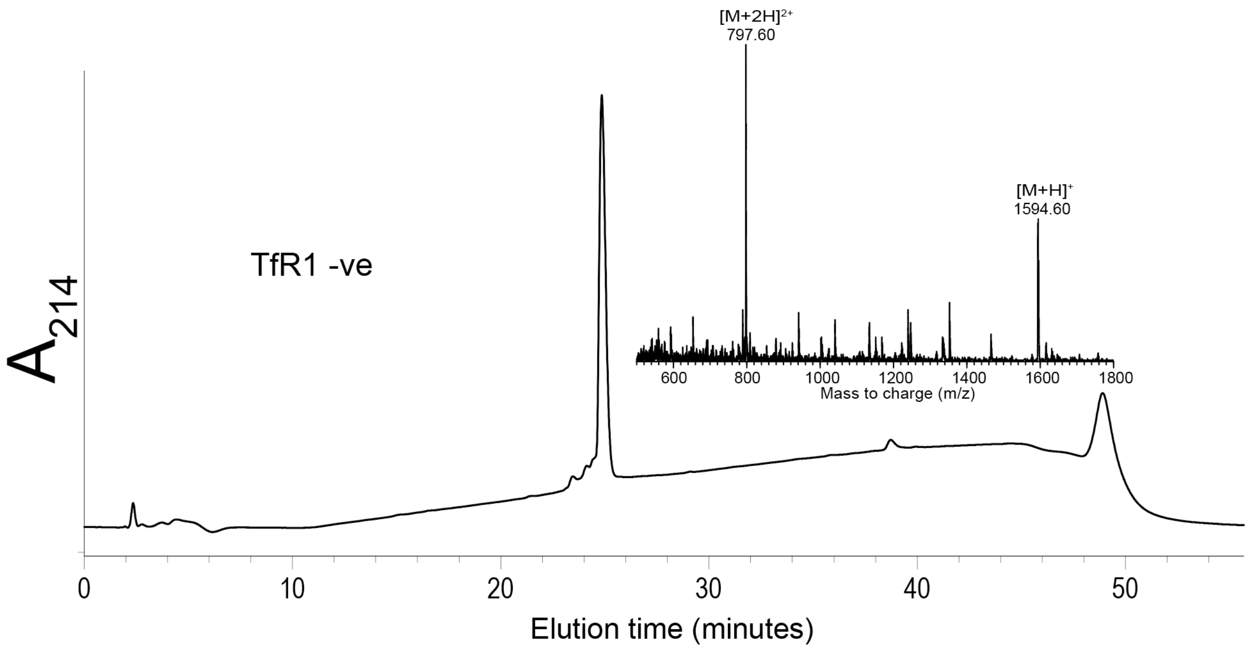

Figure S4. Analytical HPLC trace of TfR1 negative control with attached ESI-MS spectra. Analytical HPLC was run at an increasing gradient of 2% buffer B (90% ACN, 0.05% TFA) in buffer A (0.05% TFA) per minute using an Agilent, 300Å, 5 μm, 150 x 2.1 mm C18 column.

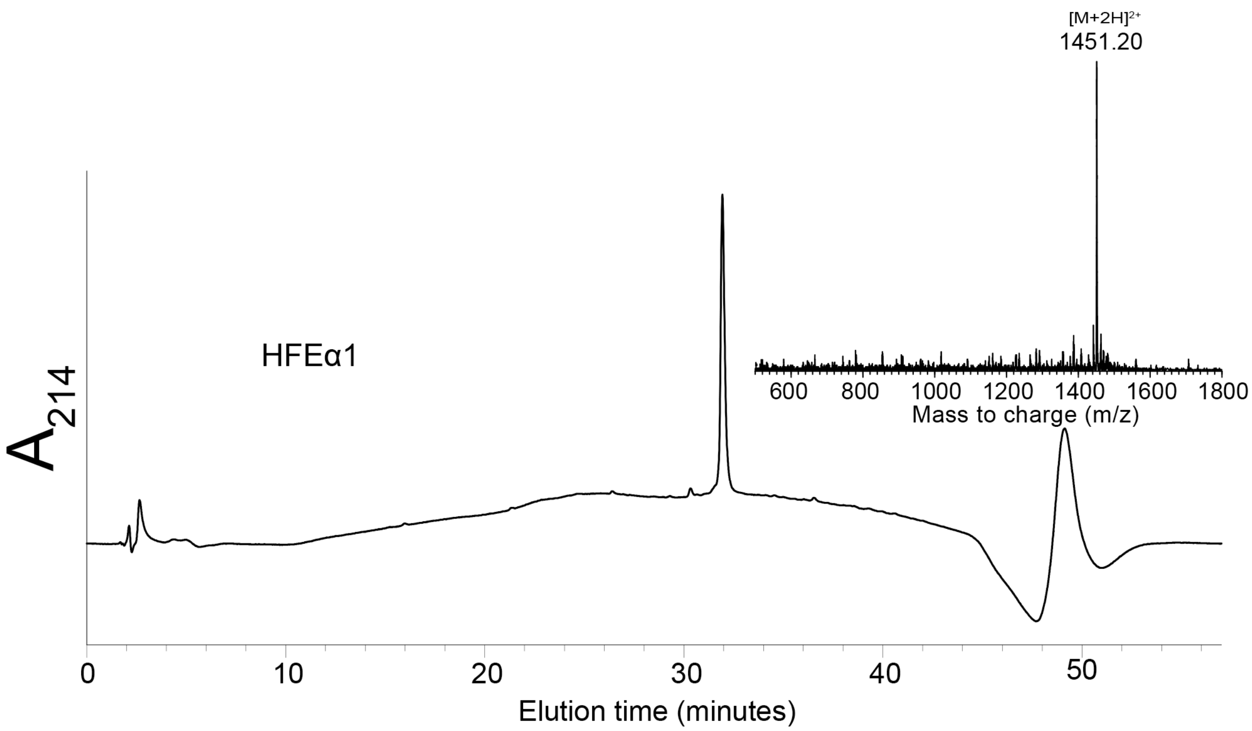

Figure S5. Analytical HPLC trace of HFEα1 with attached ESI-MS spectra. Analytical HPLC was run at an increasing gradient of 2% buffer B (90% ACN, 0.05% TFA) in buffer A (0.05% TFA) per minute using an Agilent, 300Å, 5 µm, 150 x 2.1 mm C18 column.

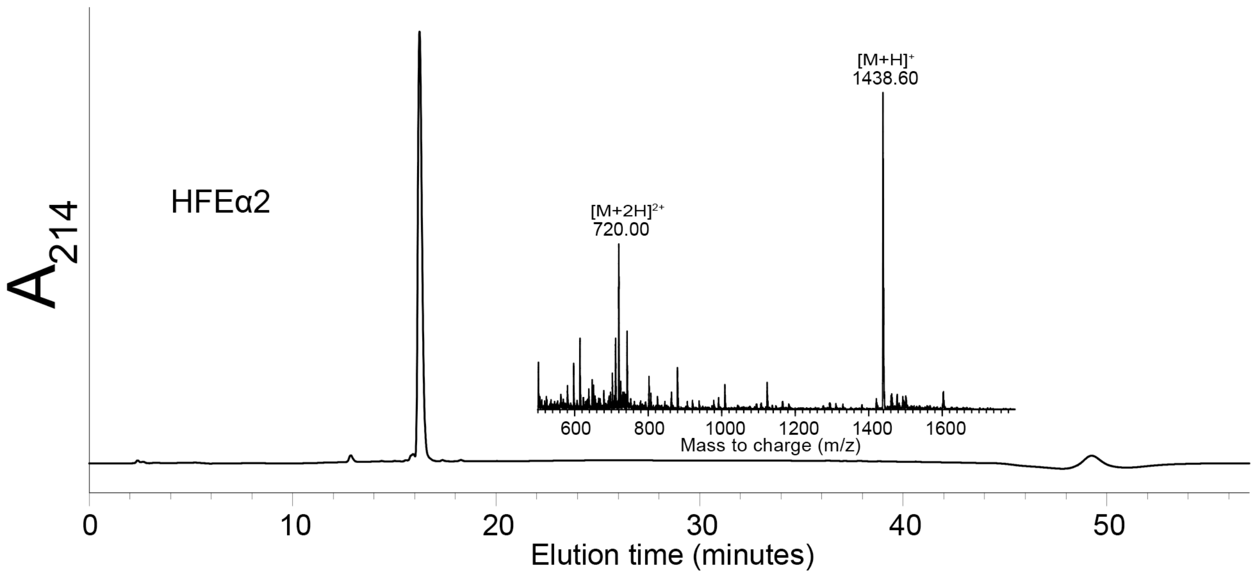

Figure S6. Analytical HPLC trace of HFE*α*2 with attached ESI-MS spectra. Analytical HPLC was run at an increasing gradient of 2% buffer B (90% ACN, 0.05% TFA) in buffer A (0.05% TFA) per minute using an Agilent, 300Å, 5 µm, 150 x 2.1 mm C18 column.

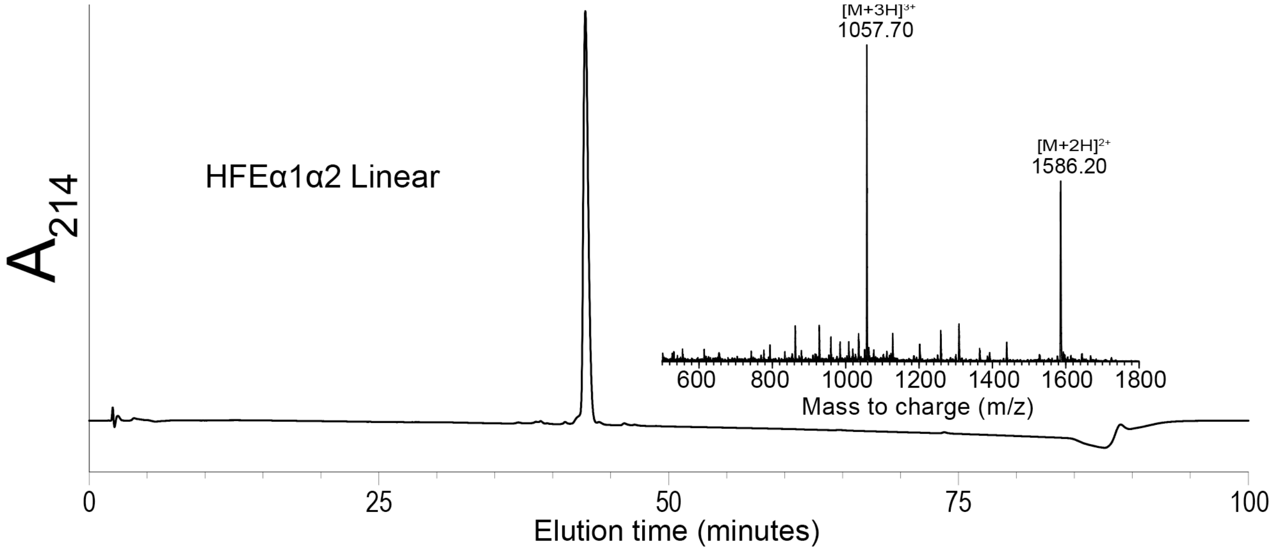

Figure S7. Analytical HPLC trace of HFE $\alpha1\alpha2$ with attached ESI-MS spectra. Analytical HPLC was run at an increasing gradient of 1% buffer B (90% ACN, 0.05% TFA) in buffer A (0.05% TFA) per minute using an Agilent, 300Å, 5 µm, 150 x 2.1 mm C18 column.

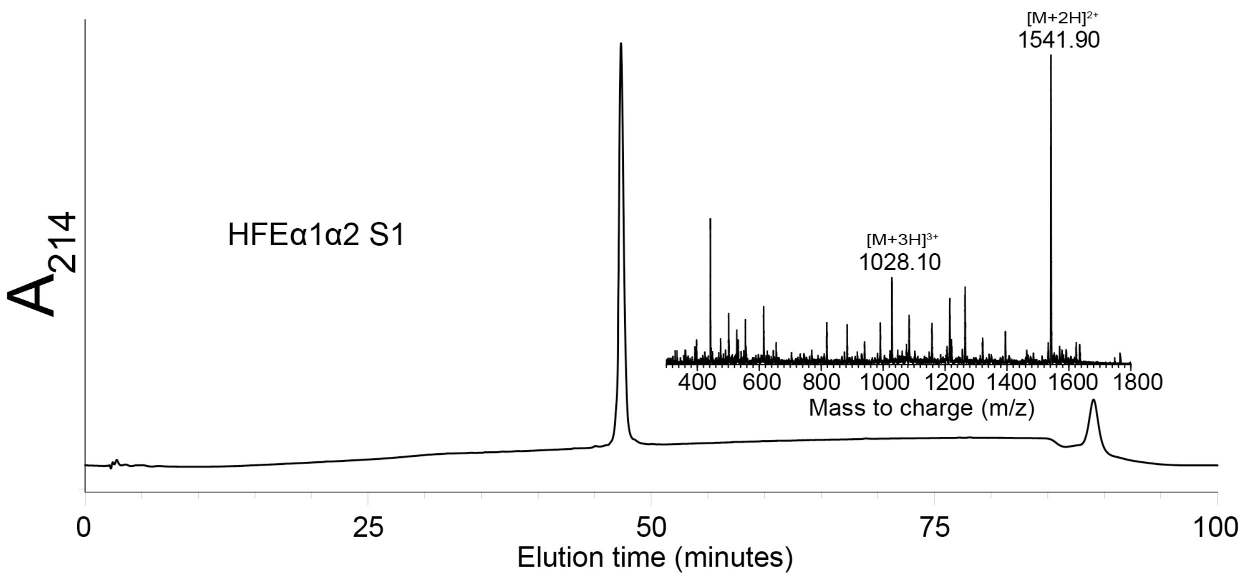

Figure S8. Analytical HPLC trace of HFE $\alpha1\alpha2$ staple 1 with attached ESI-MS spectra. Analytical HPLC was run at an increasing gradient of 1% buffer B (90% ACN, 0.05% TFA) in buffer A (0.05% TFA) per minute using an Agilent, 300Å, 5 μm, 150 x 2.1 mm C18 column.

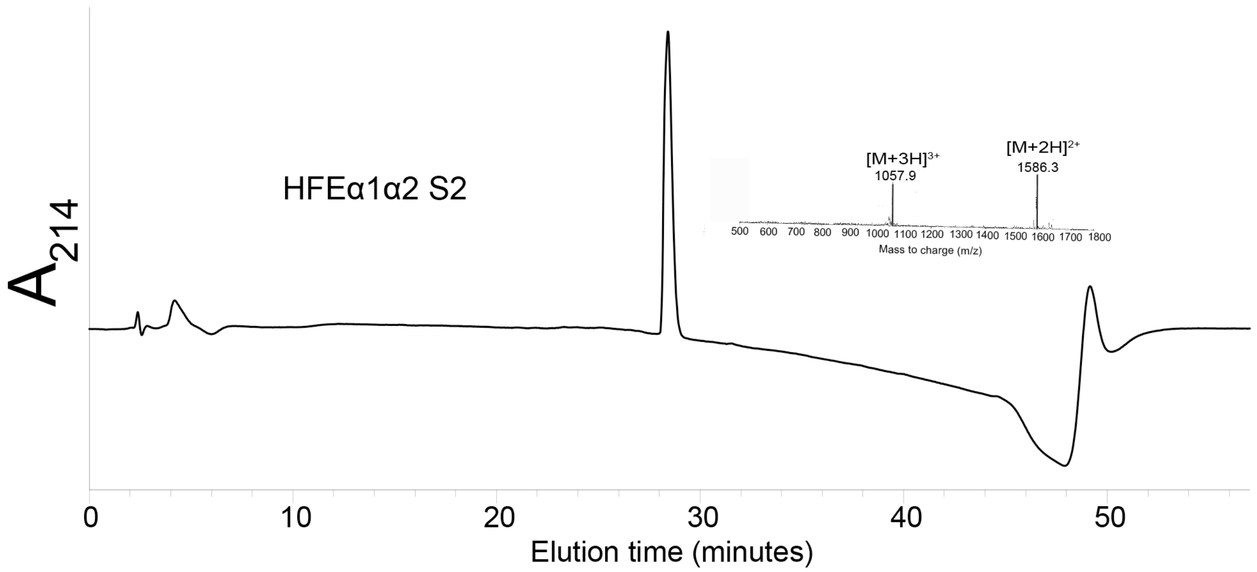

Figure S9. Analytical HPLC trace of HFE $\alpha 1\alpha 2$ staple 2 with attached ESI-MS spectra. Analytical HPLC was run at an increasing gradient of 2% buffer B (90% ACN, 0.05% TFA) in buffer A (0.05% TFA) per minute using an Agilent, 300Å, 5 µm, 150 x 2.1 mm C18 column.

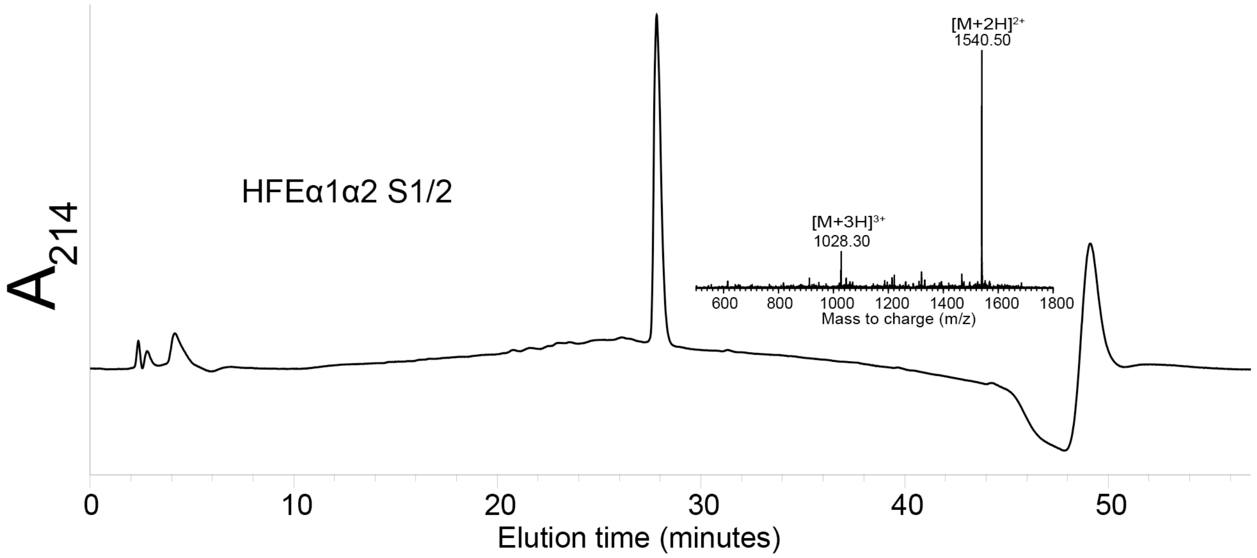

Figure S10. Analytical HPLC trace of HFE $\alpha 1 \alpha 2$ double staple with attached ESI-MS spectra. Analytical HPLC was run at an increasing gradient of 2% buffer B (90% ACN, 0.05% TFA) in buffer A (0.05% TFA) per minute using an Agilent, 300Å, 5 μm, 150 x 2.1 mm C18 column.

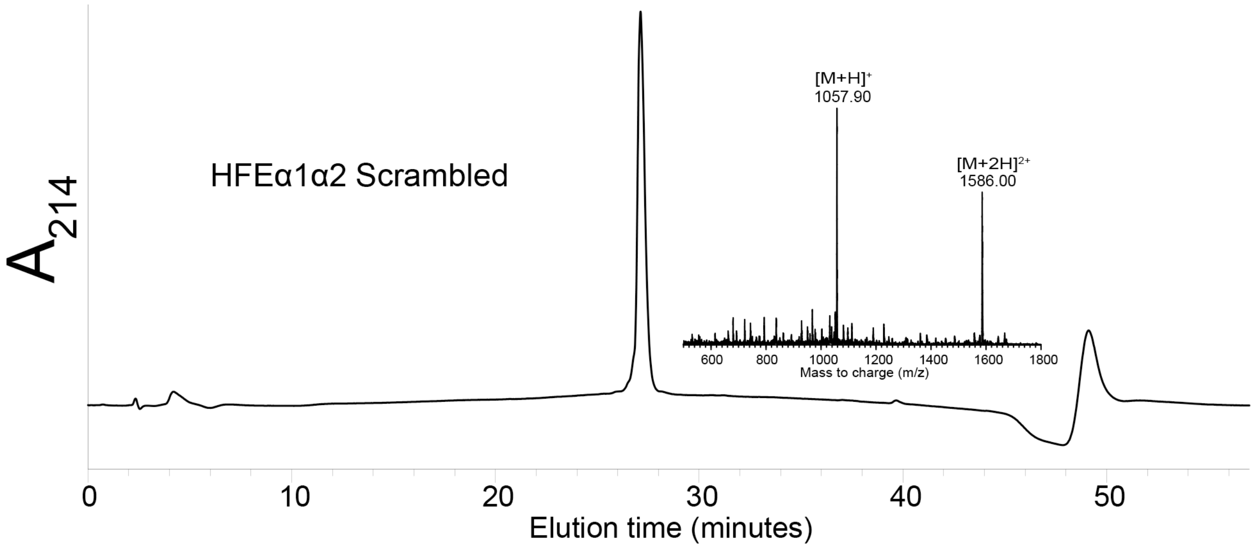

Figure S11. Analytical HPLC trace of HFE $\alpha1\alpha2$ scrambled sequence with attached ESI-MS spectra. Analytical HPLC was run at an increasing gradient of 2% buffer B (90% ACN, 0.05% TFA) in buffer A (0.05% TFA) per minute using an Agilent, 300Å, 5 µm, 150 x 2.1 mm C18 column.

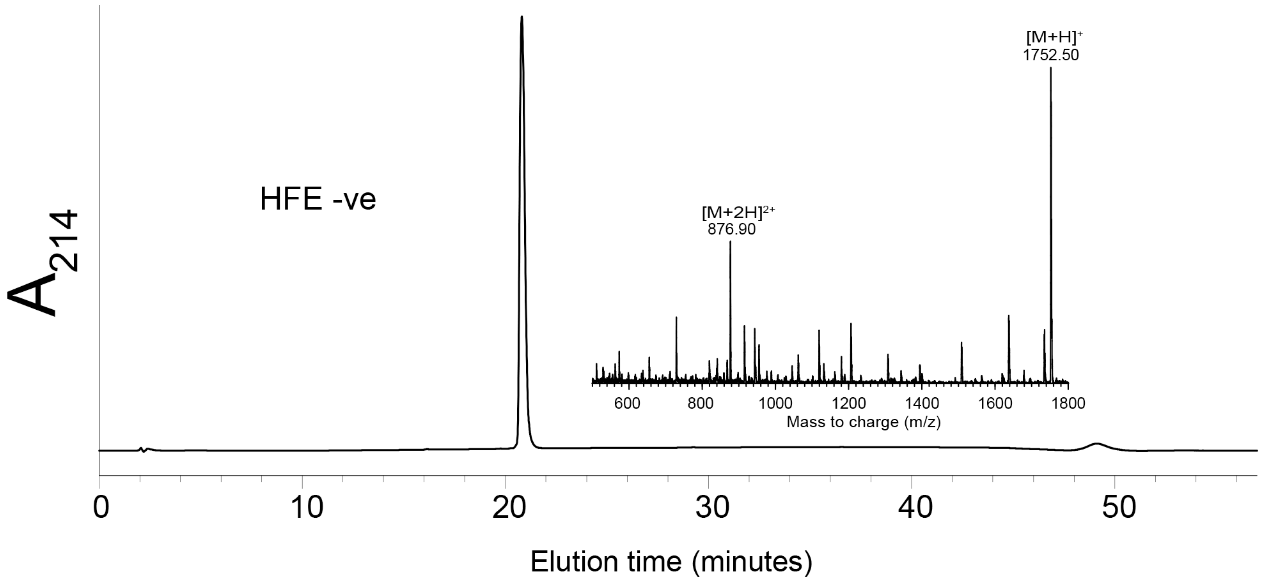

Figure S12. Analytical HPLC trace of HFE negative control with attached ESI-MS spectra. Analytical HPLC was run at an increasing gradient of 2% buffer B (90% ACN, 0.05% TFA) in buffer A (0.05% TFA) per minute using an Agilent, 300Å, 5 µm, 150 x 2.1 mm C18 column.
